# Segregate Assessment of Data Validity from the More Complex Issue of Fraud

**DOI:** 10.1017/jme.2025.42

**Published:** 2025

**Authors:** Garret A. FitzGerald

**Affiliations:** Institute for Translational Medicine and Therapeutics, Perelman School of Medicine, University of Pennsylvania, Philadelphia, PA, USA

**Keywords:** Data, Validity, Fraud, Trust

## Abstract

Trust in the validity of published work is of fundamental importance to scientists. Confirmation of validity is more readily attained than addressing the question of whether fraud was involved. Suggestions are made for key stakeholders - institutions and companies, journals, and funders as to how they might enhance trust in science, both by accelerating the assessment of data validity and by segregating that effort from investigation of allegations of fraud.

## The Background

Science depends on hypotheses, which may contain elements of truth. Hypotheses are tested using experiments that may fail to reject the absence of these truths, thereby strengthening the next hypothesis. In turn, if not rejected by the results of more experiments, the repetitive formulation of hypotheses allows more elements of truth to be assembled, like mosaics, until a picture of reality begins to emerge.

The beauty and importance of that reality depends on the ingenuity of the hypotheses that are framed by scientists and the validity of their experimental results. Even clever ideas may be wrong; a beautiful hypothesis slain by an ugly fact.^1^ However, no matter. Just come up with another one. But even when a series of hypotheses are not rejected, the passage of time, which runs serially with this process, can shape its truth. The precision of experimental measurements usually improves with time, enhancing the clarity of that emerging picture — from abstraction, through impressionism to expressionism and ending in realism.

While this is the essence of the scientific approach to discovering truth, it is also well recognized that incorrect conclusions can be drawn, due to flaws in experimental methods or in the interpretation of the results they yield. A mistaken failure to reject a hypothesis may result from inappropriate controls, inadequate sample size, simple mistakes of interpretation or flawed methods of measurement. A fundamental strength of the scientific method is this recognition of the provisional nature of results until they have been replicated, ideally by many investigators, using different approaches, many times. Thus, *the most important test in science is the test of time*: the validation of one’s discoveries by others, gradually widening their acceptance as truth. This point is often missed in public discourse, most recently during the COVID-19 pandemic when scientists were criticized for inconstancy when they revised their public health guidance on masking, social distancing and disinfecting fomites as more evidence on viral transmission emerged.^2^ As Paul Samuelson summarized John Maynard Keynes’ thinking, “When the facts change, I change my mind. What do you do, sir?” In the public square, this feature of the scientific method is poorly understood, never mind the concurrent competition from “alternative facts.”

## The Problem

While replication is fundamental to the integrity of a scientific discovery, a perception has emerged of a “replication crisis” in biomedical science. Attention was broadly drawn to this issue by publication of efforts by the private sector to recapitulate scientific results published in the literature, often from academic institutions.^3^

Pharma and biotech have a strong motivation to try and replicate published work before investing in drug discovery and development based on such foundations. It is a logical and common deployment of their resources. Such internal studies are rarely subjected to the rigor of peer review and emerge in the literature. Besides commercial competitors, this puts the biomedical community at large and arguably decision-making within that company at a disadvantage. Thus, failure to replicate may also result from a failure of the replicator. In the COX-2 inhibitor saga, early replication by the sponsors of our studies showing depression of prostacyclin biosynthesis, which we suggested might be predictive of a potential cardiovascular hazard, were never published.^4^ Thus, acceptance of this reality was postponed while the sponsors argued publicly against the validity of our observations.

Academic investigators lack resources for replication studies. A recent survey of academic investigators reported a lack of funding, institutional priority and competitiveness for publication of replication studies compared to those addressing a novel hypothesis.^5^ In the rare situations when replication studies are performed by academic investigators and accepted for publication, this lack of priority can delay publication of such “negative results” — in this case by 18 months.^6^ Thus, a failure to replicate claims about so called “pro-resolving mediators” (SPMs) published in 2015 was essentially ignored and when the methodology underlying the original claims was shown to be flawed nine years later, the emergence of that paper took 18 months from the time of submission.^7^ These features reduce the incentive of investigators to embark on such studies. The delays and difficulties in publication may jeopardize their career advancement, despite their ultimate validation with the passage of time.

Publication bias exists in various forms. These include the bias towards “positive” results in studies funded by industry^8^ and the bias of journals to avoid or delay papers that are negative. The most impactful journals are particularly on the lookout for novel, newsy discoveries.^9^ While the incentives not to publish papers that question their own previously published studies are clear, we lack the data to determine whether this is also a problem.

Leaders of academic institutions are also not motivated to address the issue of scientific validity in a transparent way. The institution may have both fiscal and reputational investments in the experimental outcome.^10^ Institutional hesitancy may also relate to the conflation of validity with fraud. The community at large is concerned in the first instance with validity — whether they can believe a set of published results — rather than, if they cannot, the reasons that lie beneath. However, such concerns with validity then raise questions about the rest of that author’s portfolio. Pursuit of such broader concerns of systematic misconduct are more time consuming and blend into consideration of outright fraud. We lack hard data on the prevalence of invalid data in the literature or of the frequency of fraud.

Just as journals may fear for their reputations being tarnished by evidence that invalidates their published papers, academic institutions are wary of the issue of fraud. Clearly, the responsibility to investigate this issue in a fair and rigorous manner and to apportion sanction rests with them. Those investigating such cases bear a responsibility to the accused, who is innocent until proven otherwise, to the whistleblower, and to their institution.

Such investigations usually involve a stepwise process — first by the relevant internal faculty committee, then by a broader committee that may include outside experts with domain expertise and finally, the decision by a separate body on how to proceed with respect to potential sanctions based on their findings.

Institutions vary as to their criteria for progression through this process, when to inform funders of the existence and progress of such an investigation (whether this is obligated varies between countries, although in the US, investigations of National Institute of Health-funded studies must be reported to the Office of Research Integrity (ORI)), whether to inform the whistleblower of the results of the scientific investigation and how to decide on sanctions. *Both funders and journals tend to cede their decisions to the outcome of these institutional investigations despite their inconstancy and lack of transparency.*

These processes are not only variable and non-transparent but are extremely slow. Anecdotal evidence shows how cautiously institutions approach these issues. Recently, investigative journalism by a TV station and a student newspaper pushed the Karolinska Institute and Stanford University respectively to broaden their investigations of research integrity. Both institutions then revised their initial conclusions, leading to the departures of their Dean and President respectively.^11^ A claim of a faculty member’s exoneration after consideration of a large portfolio his papers^12^ was removed from the website of Queen Mary’s University of London only after publication of the actual conclusions of their own investigative committee of experts that upheld all concerns of the whistleblowers.^13^ An alleged systematic pattern of scientific misconduct over decades at both the University of California San Diego and the agency by an influential leader in the National Institutes of Health has recently been reported.^14^

## Some Solutions

This issue of ensuring scientific truth has many stakeholders, each with their own incentives, budgets and investigative powers. The approach has been highly variable, lacking transparency. It is inadequately prioritized and therefore, resourced. Addressing it is thus highly complex and requires the major players — academic institutions, journals, funders and industry — to act both collectively and in concert and for each seriously to up their game. This is a big ask. However, our procedures for assessing the quality of the biomedical literature are not fit for purpose.

A few suggestions:

- Segregate the approaches to questions of fraud and validity.^15^ This is perhaps the most fundamental recommendation. Implementation of the recommendations below will let us get quickly to the question posed most commonly by the biomedical community — do I have reason to doubt the validity of the data in the paper I am reading? Institutions, journals and funders each have distinct obligations to answer that question. By contrast, answering a tougher question — was fraud involved? — is a more complex one to address for a narrower audience, is led by the employer and is more prolonged, usually taking years.

### Validity

- Publish studies that fail to replicate. As these are mostly performed by industry and the information is pre-competitive, an **Industry Led Consortium** should fiscally support and encourage publication in an open access, critically reviewed, journal or website, specifically designed for this purpose.
- A **Consortium of Journal Editors** should adopt a policy for fast-track publication of replication studies related to papers that they have published, including responses from the original authors. While the primary responsibility of a journal is to its readers, they are also responsible to reviewers who serve often highly profitable publishers, such as Elsevier and Wiley, uncompensated in the expectation of data validation.
- **Accelerate retractions or statements of concern** — after failure to replicate, this decision is often left to the institution that in turn requests it of the author. This “poacher turned gamekeeper” approach is flawed. This responsibility should revert to journals independent of institutional investigations and decisions should be made rapidly and transparently. Journals should assume their responsibility as propagators of truth.
- **Academic Promotional Committees** should build into their guidelines the importance of replication studies.
- **Data sharing** — if institutional policy requires data sharing as an assurance of validity, they should enforce it.
- **Rapid triage of “simple mistakes” from potential research misconduct**. Establish transparent ground rules for making this decision without bias at the earliest stage of an institutional investigation.
- **Funders** should provide competitively awarded grants to academic investigators to perform replication studies. Recent initiatives^16^ should be expanded.
- **PubPeer,** a curated online discussion site where largely anonymous commentators raise questions about data validity, provides a useful service as long as the tenor of the discourse remains respectful. Flagging PubPeer discussions in PubMed has been helpful in alerting investigators to such discussions. However, it has about it some of the secretive aspects of the Star Chamber.^17^ Consider removal of anonymity from its discussion as part of a broad-based approach to the issue of validity.

### Fraud

- The responsibility to address the possibility of fraud is with the academic institutions or the relevant company if an industry-based scientist is suspected of contributing fraudulent data to the literature.
- **Science Funders** should adopt policies that resource their own independent internal investigations of potential misconduct — the ORI has the right to launch its own investigations independent of institutions but is grossly underfunded to do so.
- There are many stakeholders, among them the relevant scientist, their associates, the institutions, the funders, investors in spin out companies, patients if clinical trials are involved and the public. *Prioritize a transparent and common process; this currently does not exist.*
- First a **Consortium of Academic Deans** needs to establish a common, stepwise process that protects the accused and the whistleblower but informs the latter of the scientific findings of the investigation with the option for response. Second, there should be a tiered, transparent process of investigation and sanction with institutional discretion as to what tier is determined by the findings. These benchmarks could then be published as proposed standards for endorsement by national and international academic bodies.

## Conclusion

The integrity of biomedical science rests on the validity of published data. Our current approaches to addressing concerns about validity are inconstant, inefficient, conflicted and flawed.

Concerns about fraud are more complex. They should be dealt with separately and rest with the home institution (or company) of the investigator. Again, our approaches are inconstant, potentially conflicted and non-transparent. The process here is delayed by legal considerations. However, we should remember how religious institutions felt that betrayal of trust was an internal matter better investigated and sanctioned without transparency.^18^ This did not end well.
